# Airborne Influenza A Virus Exposure in an Elementary School

**DOI:** 10.1038/s41598-020-58588-1

**Published:** 2020-02-05

**Authors:** Kristen K. Coleman, William V. Sigler

**Affiliations:** 1 Programme in Emerging Infectious Diseases, Duke-NUS Medical School, Singapore, Singapore; 2 Department of Environmental Sciences, University of Toledo, Toledo, Ohio USA

**Keywords:** Molecular biology, Epidemiology, Influenza virus

## Abstract

Influenza contributes significantly to childhood morbidity and mortality. Given the magnitude of the school-aged child population, a sizeable proportion of influenza virus transmission events are expected to occur within school settings. However, influenza virus activity in schools is not well-understood, likely due to our limited ability to accurately monitor for respiratory viruses without disrupting the school environment. In this study, we evaluated the use of a bioaerosol sampling method to noninvasively detect and quantify airborne influenza A virus (IAV) densities in a public elementary school. Air samples were collected from multiple locations in the school, two days per week, throughout an eight-week sampling period during influenza season. Real-time RT-PCR targeting the IAV M gene revealed detectable IAV on five occasions in densities ranging from 2.0 × 10^−1^ to 1.9 × 10^4^. No significant differences in IAV densities were related to student presence/absence. The majority of IAV-associated particles were ≤4 μm in diameter, and theoretical calculations indicate infectious thresholds after minutes of exposure. Our study represents the first identification and quantification of airborne influenza virus in an elementary school, and the results suggest that airborne IAV has the potential to circulate in schools during influenza season, in large enough doses known to cause infection.

## Introduction

Monitoring pathogen exposures in the environment is a longstanding practice to predict and investigate the incidence and spread of infectious diseases. Schools, in particular, have been identified as sources of influenza outbreaks^1–4^, and monitoring influenza-like illness in the school-aged population is a current strategy for predicting communal influenza^5^. In both seasonal and pandemic influenza outbreaks, schoolchildren are often the first to become ill^1,4,6^. Furthermore, modeling studies suggest that the highest incidence of respiratory infection is among school-aged children and young adults who contribute significantly to the spread of infections during the early stages of emerging respiratory epidemics^7^. In some communities, school-centric data, such as the number of student absences (total and those due to illness) and school nurse visits, are recorded and used for early detection of influenza outbreaks^5^. However, due to the inconsistency of self-reported data, the use of student health reports to predict influenza outbreaks is not a standardized practice. Therefore, the development of an effective method to monitor influenza virus in schools is necessary to enhance efforts to manage student and community health.

Human behaviors and their environments influence respiratory virus disease susceptibility, severity, and transmissibility^8^, especially among children. Specifically, the high incidence of influenza in children is thought to be related to interactions with schoolmates and siblings^6^, as pathogen transmission is directly related to host proximity and density^9^. Influenza virus is transmitted through (i) direct contact with infected individuals and their respiratory secretions, (ii) indirect contact with infectious secretions via surfaces or fomites such as keyboards, toys, and doorknobs, and (iii) inhalation of virus particles^10–12^. Although contact transmission plays a key role in influenza transmission^13^, improved methods for studying aerosolized respiratory viruses have improved our understanding of airborne exposure risks^14–18^. A single infectious sneeze can result in 40,000 aerosolized droplets^19^, and when expelled, can travel nearly 2 meters before falling to the nearest surface, or can evaporate^20^, resulting in droplet nuclei that can persist in the air for up to 30 hours^21^ and cause severe, lower respiratory tract infections among adults and children^22–24^. Airborne transmission can account for approximately half of all household influenza A virus transmission events^25^, while the virus exhibits 20-fold higher infectivity through inhalation than intranasal inoculation^26^, obviating the significance of illness risks associated with airborne influenza virus.

Several factors support the development of a mechanism to monitor viral activity in schools. Influenza infects approximately 20–30% of children each year^27^ and contributes significantly to childhood morbidity and mortality^28,29^. Children in the U.S. spend 13 years completing their primary and secondary education, resulting in approximately 15,000 hours spent in school facilities^30^. Therefore, a sizeable proportion of influenza virus transmission is expected to occur in the school setting, especially since compromised indoor air quality impacts 50% of U.S. school facilities^31^. Student attendance is also affected, as influenza infections cause an annual loss of 38 million school days in the U.S.^32^. The impacts also extend beyond schoolchildren, resulting in parental work loss and decreased productivity^33^.

Available evidence suggests that high densities of airborne influenza virus circulate in schools, and efforts to address negative health and community impacts center on understanding influenza transmission routes. Unfortunately, the ability to directly monitor airborne viral densities in schools lacks methodology that is efficient, sensitive and nondisruptive to students. Therefore, the goal of this study was to (i) noninvasively detect airborne influenza virus in a public elementary school, (ii) identify influenza virus transmission “hotspots” by comparing airborne influenza virus densities collected from different locations inside the school, and (iii) gain insight on the theoretical severity of illness resulting from particle exposure by determining the sizes of virus-laden particles and their relationship to measurable environmental variables (i.e., temperature, humidity, and student presence/absence).

## Results

A total of 128 air samples were collected over an eight-week period (February–March), followed by qRT-PCR targeting the influenza A virus (IAV) M gene, which revealed detectable IAV in 5% (5/96) of the air samples collected indoors, in densities of 2.0 × 10^−1^, 1.9 × 10^3^, 3.8 × 10^3^, 1.5 × 10^4^ and 1.9 × 10^4^ M gene copies m^−3^ air (Table 1). IAV was not detected in any of the outdoor reference samples. An R^2^ value of 0.97 for each standard curve was achieved for the qRT-PCR assays and the detection limit for IAV M gene was 1 RNA copy per reaction volume (25 μL) when the cut-off for positive result was set at 40 cycles. Because each influenza virus particle packages one copy of the M gene^34^, our results can also directly reflect the number of virus particles m^−3^ air. However, our results are reported in *copy numbers* m^−3^ air to remain consistent with other quantitative studies measuring airborne IAV densities.

**Table 1:** Airborne influenza A virus (IAV) densities and virus-laden particle sizes in a school.

Sampling week	Julian day	Airborne IAV densities (M gene copies m^−3^ air)*	Location	Time	Virus-laden aerosol/particle size	Exposure time needed to initiate infection^†^
1–6	35–73	ND	—	—	—	—
7	77	0.2	Gym	PM	>4 μm	Insufficient^‡^
	80	3,800	Gym	AM	>4 μm	3 mins
	80	1,900	Classroom	PM	<1 μm	6 mins
8	84	15,200	Corridor	AM	1–4 μm	1 min
	87	19,000	Corridor	AM	1–4 μm	1 min

ND = none detected.

*1:1 relationship to virus particles m^−3^ air, based on evidence that one influenza virus particle packages only one copy of the M gene^34^.

^†^Based on calculations in Table 2.

^‡^Insufficient amount of virus detected to theoretically induce infection.

Significantly different (p = 0.049) airborne IAV densities were detected between all three indoor locations (i.e., gymnasium, classroom, and corridor) and all positive samples were collected during the last two weeks of the study (month of March, Table 1). Sample location was strongly correlated with IAV density (r^2^ = 0.95), with the highest density of IAV detected in the corridor. Two (6%) of the air samples collected from the gymnasium were positive for IAV, as well as two (6%) from the corridor, and one (3%) from the classroom. No significant difference in IAV densities was observed between samples collected during and after school (p = 0.14). Indoor relative humidity (RH) and temperatures remained relatively consistent (19–29% RH and 20–23 °C) throughout the study. No significant difference in IAV densities was observed between samples collected at different RH levels (p = 0.74) or temperatures (p = 0.10).

Significantly different (p = 0.049) airborne IAV densities were detected among particle size fractions. Particle size was strongly correlated with IAV density (r^2^ = 0.95), with 91% of virus-laden particles detected in respirable size fractions (≤4 μm in diameter). The largest virus-laden particles (>4 μm, 9% of total particles) were detected only in the gymnasium, while the smallest (<1 μm; 5% of total particles) were detected only in the classroom. No significant difference in virus-laden particle size was observed among samples collected at different RH levels (p = 0.82) or temperatures (p = 0.44), nor during and after school hours (p = 0.44).

Theoretical exposure threshold calculations accounting for body mass, tidal volume, and breathing rate predict that exposure to an equivalent of 1.1 × 10^4^–2.8 × 10^5^ IAV RNA copies m^−3^ air for one minute is sufficient to induce infection in a student (Table 2). Based on the airborne IAV densities detected in the school, we estimated that students in the classroom (Day 80), gymnasium (Day 80), and corridor (Day 84 and 87) were at risk of infection following 6, 3 and 1 minute(s) of breathing, respectively (Table 1).

**Table 2:** Airborne influenza A virus exposure times and densities needed to initiate infection.^*^

Airborne exposure time	PCR-detectable gene copies^†^ m^−3^ air
1 min	11,161–281,250
2 mins	5,581–140,625
3 mins	3,720–93,750
4 mins	2,790–70,313
5 mins	2,232–56,250
6 mins	1,860–46,875
7 mins	1,594–40,179
30 mins	372–9,375
45 mins	248–6,250
1 hour	186–4,688
2 hours	93–2,344
3 hours	62–1,563
8 hours	23–586

^*^Based on the airborne infectious dose (0.6–3.0 TCID_50_) for the influenza laboratory strain A/California/04/2009 (H1N1) as measured by Alford *et al*.^66^, and a 20–30% relative humidity level; Descriptive of an average elementary school student in the USA weighing ~23–32 kg with an assumed tidal volume (V_T_) of 7 mL per kg of body mass.

^†^Irrespective of IAV target gene; Based on the assumption that one TCID_50_ is equivalent to ~300 PCR-detectable IAV RNA copies^47–50^.

## Discussion

Aerosolization is an important mechanism for spread of IAV^25^. Since children are significantly burdened by influenza and play a key role in transmission^5,29,35,36^, we established a protocol through which IAV densities could be monitored in a school setting. Viral densities were compared to theoretical IAV exposure thresholds, above which students are expected to become infected. Student illness and absenteeism caused by respiratory diseases is thought to occur, in part, because of prolonged time spent in school buildings, 50% of which experience impaired indoor air quality^31^. Therefore, understanding how the student environment influences disease transmission can not only help to better address student health, but also improve sanitation/cleaning practices and inform predictive efforts. To our knowledge, our work represents the first identification and quantification of airborne IAV in an elementary school, showing that schools can not only harbour airborne IAV densities similar to those found in clinical settings^37,38^, but also in infectious doses. Furthermore, the detection and quantification of airborne IAV in the school airshed warrants future investigations to determine the relationship between IAV densities and student illness.

Three locations in an elementary school were sampled for airborne IAV, including the main corridor, gymnasium, and a classroom. Because each location featured different IAV densities and particle sizes, the exposures of students to IAV differed according to sample location. For example, students were likely at an elevated risk of IAV infection by breathing in the classroom on Day 80, especially since the particles were <1 μm in diameter. Infection could have also arisen from breathing for 3 minutes in the gymnasium on Day 80, but is less likely, as particles were >4 μm in diameter. While no specific activity can explain elevated IAV densities in the classroom, two defined activities in the gymnasium throughout the school day are thought to promote elevated IAV densities. First, the gymnasium is used by approximately 25 students during each class period for physical education instruction. Physical activity/exercise can result in aerosolized IAV through increased respiratory rates, and increased respiratory distress due to bronchoconstriction^39^, which has been demonstrated to affect up to 16% of children^40^. Second, the gymnasium hosts the student lunch period, which lasts for 50 minutes, during which two cohorts (approximately 235 students each) eat for 25 minutes. The entire student body is not only represented in the gymnasium during the lunch period, but is also moving, en masse, as each cohort enters and exits the space, theoretically creating turbulence to maintain suspension of IAV (if present) throughout the period, and likely into the post-lunch hours.

Airborne IAV densities were highest in the school corridor, which was expected, and can be partially explained by two factors. First, student lockers are situated along the walls of the corridor which encourages air turbulence as students pass through the corridor and open and shut locker doors. Second, the corridor represents the only available passage between classrooms and therefore each student passes through the corridor multiple times per day, which dually increases the probability that an infectious student will shed virus in the area, and that other students will be exposed. In a college student office setting, Zhang and Li (2018)^41^ demonstrated that the frequency of close contact (within 1 m) is 9.64 contacts per hour per student, which contributed to 45% of reported IAV infections. Additionally, previous studies have suggested that influenza illness and death rates could be decreased by as much as 50% by reducing the contact rates of infected persons^42^. Given the high airborne IAV densities detected in the school corridor, along with elevated student contact rates, it is plausible to conclude that the school corridor is a “hotspot” for influenza virus transmission.

Studies focusing on clinical environments have demonstrated that a considerable proportion (48–53%) of total airborne IAV-laden particles are respirable^37,38^. In the current study, the majority (91%) of airborne particles associated with IAV were respirable (≤4 μm in diameter), representing the most infectious fraction based on size. Particles >4 μm in diameter are deposited predominantly in the nasal cavity or trachea, and are subject to mucociliary clearance before initiating infection^43^. In contrast, particles ≤4 μm in diameter can be deposited deep into the lungs, and are more likely to result in lower respiratory tract infections, which disproportionately impact children during influenza pandemics^24^. Therefore, identifying the particle size distribution of airborne IAV is critical for understanding the potential transmission and infectious impact of the virus.

Although we successfully detected airborne IAV in the school with appropriate sensitivity to accurately quantify IAV densities, environmental factors created sample processing challenges and difficulty interpreting the data. Airborne pathogen densities in nonclinical environments can be several orders of magnitude lower than those detected in clinical environments^37,38,44^. Furthermore, desiccation stress is known to limit the stability of airborne IAV^45^. Therefore, in the school, lower IAV densities and high variability were anticipated, and sampling parameters were chosen to maximize detection, including short sampling durations to preserve the integrity of captured IAV RNA. We chose a flow rate of 3.5 L/min for four hours at a time, which was previously demonstrated to efficiently capture and preserve airborne IAV RNA for RT-PCR detection^37,38^. Next, IAV packages a single copy of our RNA target, the M gene^34^, and therefore a 1:1 relationship between the number of gene copies detected and number of virions is a valid assumption. However, Brown *et al*. (2015) indicated that IAV densities can be overestimated when quantifying gene copies^46^, and studies have assessed the relationship between RNA copy number and the number of viable viruses, suggesting that one TCID_50_ is equivalent to ~300 PCR-detectable IAV RNA copies^47–50^. While IAV viability was not directly assessed in the current study, our quantitative results do provide an estimate of the potential for IAV transmission in the school environment, consistent with the prevailing public health proposals for conservative estimates of disease transmission risk^51^.

Lower densities of airborne IAV were expected in the absence of students from the building. However, no significant difference in airborne IAV density, as a function of student presence or absence, was observed. This result was unexpected, as children are thought to be key vehicles of IAV transmission and are viewed here as the major factor contributing IAV to the school airshed. We did not collect individual student health data during the study, and therefore could not definitively link the prevalence of influenza among the student population with virus detections. However, the detection of IAV during the absence of all students from the building was nonetheless an important observation, indicating persistence of the virus in the airshed. Airborne IAV can remain viable for up to 36 hours^52,53^ and is likely facilitated by two factors. First, it has been demonstrated that temperature and RH influence the viability and transmission of influenza viruses^54–58^. However, research has recently demonstrated that RH does not influence influenza virus viability^59^, but rather the rate of aerosol deposition, which influences the concentration of virus particles in the air. In the current study, the environmental conditions during the school day (19–29% RH and 20–23 °C) were optimal for IAV persistence. Second, children shed IAV for a longer duration than adults shed the virus^60^, encouraging prolonged IAV aerosolization in a school setting where children predominate. Overall, our findings demonstrate that on the short term, IAV is not fully cleared from the school environment upon removal of students, and support the assertion that schools should be considered an IAV transmission hotspot, even in the absence of students.

Although airborne influenza virus has been detected in select indoor settings^37,38,44,61–65^, the ability to consistently detect the virus in the airshed remains limited in environments featuring low IAV densities. We now have the first molecular evidence of airborne IAV in an elementary school, during a portion of the influenza season when students were exposed for appropriate durations to densities of influenza-laden particles that could facilitate infection. Furthermore, given the magnitude of the school-aged population, our data provide justification for considering schools as influenza hotspots, warranting further study to determine the relationship between airborne IAV densities and student health to improve influenza management in the greater community.

## Methods

### Bioaerosol sampling

Air samplings were performed four times per week during an eight-week sampling period (February–March) in an elementary school (Toledo, OH area) that enrolls approximately 470 students, in grade levels K–6. Airborne IAV was sampled in the school gymnasium, a classroom, main corridor, and an outdoor reference, using two-stage bioaerosol cyclone samplers provided by the National Institute for Occupational Safety and Health (NIOSH) and chosen for their portability, durability, minimal preparation time, and efficiency that equals that of commercial samplers^14^. Each sampler was placed 1.2 m from the ground, simulating the average elementary school student’s breathing level, and connected to an SKC AirChek XR5000 pump (SKC, Eighty Four, Pennsylvania) with 6.35-mm Tygon tubing, operating at a flow rate of 3.5 L of air min^−1^, collecting a total of 840 L of air for each sample. The pump flow rate and sampling duration was based on previous studies that demonstrated efficient capture of airborne influenza virus RNA for RT-PCR detection^14,37,38^. Each sampler collects particles >4 μm in diameter into a 15 mL centrifuge tube, particles 1–4 μm in diameter into a 1.5 mL centrifuge tube, while particles <1 μm in diameter are collected onto a 37-mm diameter, polytetrafluoroethylene filter with 2-μm pores. The influence of student presence/absence on airborne influenza virus detection was determined by collecting samples early in the school day (8:00 am–12:00 pm, students present), and in the afternoon/evening (3:00–7:00 pm, students absent). After sampling, collection tubes and filter cassettes were transported to the laboratory on ice and stored at −80 °C, if not immediately processed. Samplers were washed with isopropanol and air dried between sample collections. Temperature and RH were continuously recorded inside the school building near each sampler using HOBO dataloggers (Onset; Bourne, MA, USA).

### RNA extraction and influenza virus detection

Sample RNA was extracted and purified using the MagMAX Viral RNA Isolation Kit (Ambion) following the manufacturer’s instructions with slight modifications, including the addition of lysis/binding solution directly to (i) sampler tubes, and (ii) 50-mL Falcon tubes containing the PTFE filters. Xeno RNA Control (Ambion), a synthetic RNA transcript, was added to the sample lysis solution to act as an internal, positive control target for assessing the efficiency of RNA recovery. Purified RNA was eluted in 30 μL of elution buffer. All analysis materials were purchased RNAse-and pyrogen-free, if possible, and otherwise depyrogenated by autoclaving at 250 °C for 30 minutes.

One-step, real-time, RT-PCR targeting the influenza A virus M gene was performed in an Applied Biosystems Step-One Real-Time PCR system with commercial TaqMan AIV-Matrix Reagents (Ambion) in a total reaction volume of 25 μL. Reverse transcription was performed at 65 °C for 5 min, 50 °C for 2 min, and 95 °C for 10 min, followed by 65 cycles of qPCR analysis at 95 °C for 15 s, and annealing/elongation at 60 °C for 1 min. Three negative control reactions (no template) were included in each qRT-PCR assay. To quantify the viral load present in each sample, the Ct value from each reaction was compared to those of a standard curve derived from a dilution series of known quantities of IAV M gene copies.

### IAV exposure threshold calculations

Based on observed densities of airborne IAV, we estimated the breathing time, above which a student could theoretically become infected. To calculate the time, we used a known range of 0.6–3.0 TCID_50_ (TCID_50_ is the number of IAV particles that induce infection in 50% of inoculated tissue culture cells)^66^, which has previously been used to estimate the risk of airborne IAV infection after exposures consistent with a one-hour clinical visit, an eight-hour workday, and after 24 hours indoors^44^. Since approximately 300 RNA copies is equivalent to one TCID_50_^47–50^, the resulting threshold IAV density capable of initiating an infection is theoretically equivalent to 50–900 RNA copies. Assuming the average elementary school student weighs 23–32 kg, has a respiratory rate of 20 breaths min^−1^, and a tidal volume (air volume displaced in a single breath) of 7 mL per kg of body mass, we calculated an inhalation volume range of 3200–4480 mL air min^−1^ for a typical student, which was then compared with our estimated IAV densities to determine the number of minutes of breathing necessary to cause an infection.

### Statistical analysis

Data were imported into STATA version 15.0 (StataCorp, College Station, TX, USA) and a two-sample t-test or one-way analysis of variance was performed to test for significant differences in IAV densities and particle sizes between indoor sample locations (gym, classroom, corridor) and environmental conditions (temperature, RH, and student presence). Regression analyses were then performed to test for correlations between variables demonstrated to be statistically significant.

## Data Availability

Datasets generated during this study are available from the corresponding author upon reasonable request.
